# Comparative nutritional analysis and sensory evaluation of three baby foods with low allergen ingredients for infants

**DOI:** 10.1016/j.fochx.2025.102533

**Published:** 2025-05-07

**Authors:** Nourhan M. Abd El-Aziz, Hanem M.M. Mansour, Marwa R. Elbakatoshy, Taha Mehany, Oscar Zannou, Reza Tahergorabi, Mohamed G. Shehata

**Affiliations:** a Food Technology Department, Arid Lands Cultivation Research Institute (ALCRI), City of Scientific Research and Technological Applications (SRTA-City), Alexandria 21934, Egypt; b Department of Food Technology, Vocational School of Technical Sciences at Mersin Tarsus Organized Industrial Zone, Tarsus University, 33100 Mersin, Turkey; c Food and Nutritional Sciences Program, North Carolina A&T State University, Greensboro, NC 27411, USA; d Food Research Section, Applied Research and Capacity Building Division, Agriculture and Food Safety Authority (ADAFSA), Abu Dhabi 52150, United Arab Emirates

**Keywords:** Complementary food, Nutritional value, Antioxidants, Amino acids, Essential vitamins

## Abstract

This study explores the nutritional composition and sensory acceptance of three baby food formulations designed to aid infants' transition to solid foods. The formulations were composed of different raw materials: BF1 (chickpeas, rice, artichoke, carrots, orange peel), BF2 (corn, egg white, spinach, carrots, orange peel), and BF3 (potato, mushroom, beet, carrots, orange peel). Results showed that BF1 had the highest protein content, moderate fat, and significant fiber. BF2 contained higher levels of vitamin D3 and E, while BF1 had more vitamins A and B12. BF2 contained the highest levels of essential amino acids while BF3 contained the highest amount of fatty acids. Antioxidant activity was highest in BF1, followed by BF3 and BF2. Sensory evaluations ranked BF3 the most acceptable, followed by BF1 and BF2. These results highlight differences in nutritional and sensory attributes, warranting further research into their implications for infant health, particularly at the industrial scale.

## Highlights

• New baby foods from several nutritious matrices were developed.
• BF2 contained highest tryptophan, α-linolenic &linoleic acids, vitamins D3 &E ratios.
• BF3 received the highest overall sensorial score then BF1 and BF2.
• BF1 displayed elevated levels of vitamins A &B12, linoleic acid, and antioxidant activity.
• Further research is necessary to involve these differences on infant health and growth.

## Graphical abstract

**Figure:**
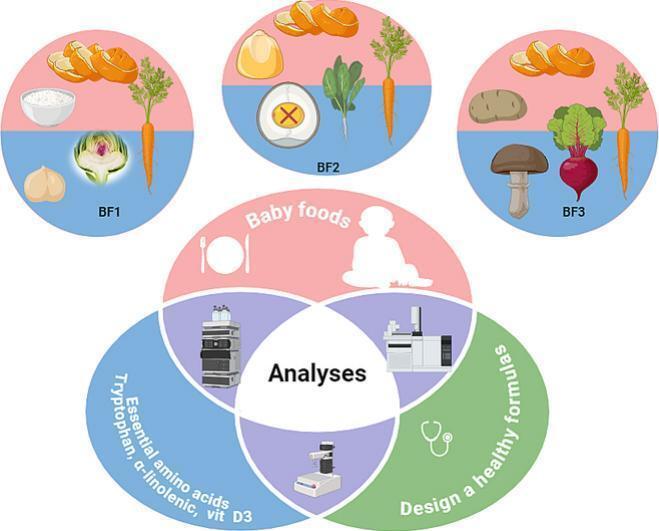

## 1 Introduction

The period between six and twenty-four months of age represents a critical window for infant growth, brain development, and the establishment of lifelong dietary patterns. During this phase, breast milk alone becomes insufficient to meet the escalating nutritional demands of the growing child. The timely introduction of complementary foods is therefore essential to fill the nutritional gap and to support healthy development (WHO, 2021). Optimal complementary feeding practices are particularly crucial in low- and middle-income countries, where inadequate nutrition during this stage contributes significantly to childhood malnutrition, stunting, and micronutrient deficiencies (UNICEF, 2020).

Exclusive breastfeeding is recommended for the first six months of life, as it provides all the energy, macronutrients, and immunological protection needed by infants (WHO, 2021). However, from six months onward, complementary foods—defined as any nutrient-rich solid or semi-solid food or liquid other than breast milk or infant formula—should be introduced to meet the evolving nutritional needs. These foods must not only be nutritionally adequate but also safe, age-appropriate in texture and consistency, and acceptable to both infants and caregivers (American Academy of Pediatrics, 2019). In most studies, early CF (<4 months) is not related to a higher risk of atopic dermatitis, food allergy, asthma, or rhinoconjunctivitis. In contrast, late CF (after 6–7 months) is associated with a higher risk of food allergy after 5 years of age10 but it is not associated with asthma, atopic dermatitis, or allergic rhinitis in the majority of studies (Hicke-Roberts et al., 2020). Beyond meeting nutritional requirements, complementary foods must be introduced with attention to allergenic potential. Food allergies in infants can range from mild cutaneous or gastrointestinal symptoms to severe anaphylactic reactions and may manifest immediately or after a delayed period (Koletzko et al., 2012; NIAID, 2020). In accordance with the Codex Alimentarius standards, the presence of major allergens—including milk, eggs, fish, peanuts, soybeans, tree nuts, wheat, and sulfites—must be clearly labeled when exceeding regulatory thresholds (FAO/WHO, 2022). For infants with allergic predispositions, tailored dietary planning and appropriate nutrient supplementation are critical to ensuring nutritional adequacy without compromising safety (Agostoni et al., 2010; WHO, 2021). Given these multifaceted considerations, the formulation of complementary foods requires the judicious selection of raw materials and processing techniques that enhance both nutritional quality and functional properties. In this study, ingredients such as chickpeas, rice, artichoke, corn, egg white, spinach, potato, mushroom, beetroot, carrots, and orange peel were selected based on their proven nutritional value—especially their richness in plant-based proteins, iron, zinc, dietary fiber, and provitamin A. These ingredients are widely available, culturally familiar, and commonly utilized in traditional weaning practices, thereby enhancing their practical applicability and acceptability in diverse communities. Sensory attributes—including color, taste, aroma, and texture—play a central role in determining the acceptability of complementary foods by both infants and caregivers. Previous research has shown that favorable sensory characteristics enhance feeding success and compliance, which are essential for maintaining adequate nutrient intake and preventing feeding difficulties (Agbemafle et al., 2020; Mahmoud & El Anany, 2014). Additionally, the rheological and textural properties of complementary foods—such as viscosity, consistency, and flow behavior—directly influence ease of swallowing, oral-motor development, and the transition from breastfeeding to semi-solid and solid foods (Steele et al., 2015). Based on these considerations, the present study aimed to develop three novel complementary food formulations (BF1, BF2, BF3) using regionally accessible, nutrient-dense raw materials. The formulations were evaluated for their proximate nutritional composition, sensory acceptability, and rheological characteristics. The findings of this study are intended to inform the development of safe, nutritious, and culturally relevant complementary food products suitable for infants during the weaning period.

## 2 Materials and methods

### 2.1 Raw materials

The raw materials of the new formulated baby foods were as follow: chickpeas, rice, artichoke, corn, egg white, spinach, potato, mushroom, beet, carrots, and orange peel. All raw materials for baby food constituents were obtained from the local markets in Alexandria city and New Borg El Arab city, Egypt in 2023. All reagents and chemicals used in the present study were from analytical grade and purchased from Fisher Scientific UK.

### 2.2 Preparation of baby foods

**Fig. 1:**
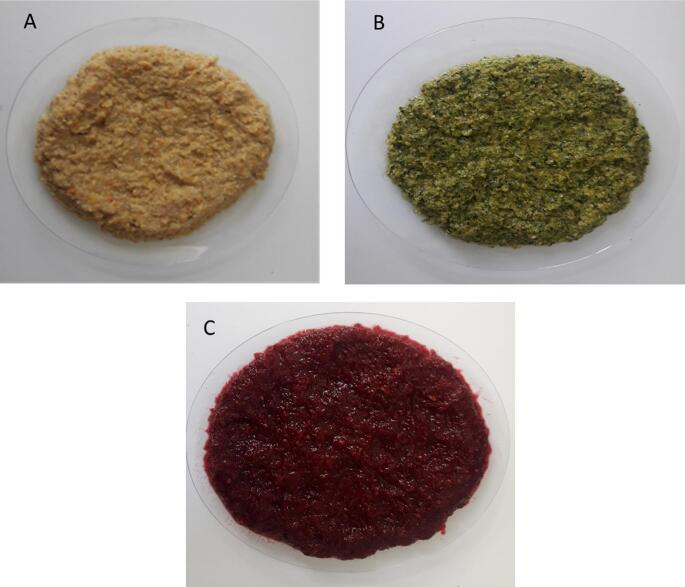
New cooked baby food formulas images. BF1: baby food 1; BF2: baby food 2 and BF3: baby food 3.

**Fig. 2:**
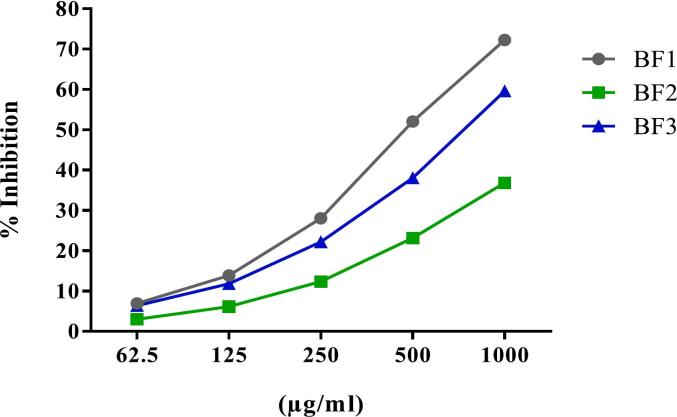
Antioxidant activity by DPPH scavenging activity of new developed baby food formulations. BF1: baby food 1; BF2: baby food 2 and BF3: baby food 3.

First, the obtained plant raw materials were dried in an oven at 50 °C for 48 h or till completely dried. Then they powdered by a mechanical grinder separately. After that, each baby food formula was mixed by the determined weight as illustrated in Table 1 for producing three different formulations, i.e., BF1, BF2, and BF3. Approximately 10 g from each formula plus 100 ml of water were cooked at 70 °C for 15 min with continuous spoon stirring. Finally, the three developed baby foods (Fig. 1) were stored at −20 °C for further analysis. (See Fig. 2.)

**Table 1:** Average weight of new baby food constituent.

Constituents (g)	BF1	BF2	BF3
Chickpeas	15.88	–	–
Rice	12.05	–	–
Artichoke	5.504	–	–
Corn	–	18.4	–
Egg white	–	3.64	–
Spinach	–	0.63	–
Potato	–	–	15.6
Mushroom	–	–	3.2
Beet	–	–	2.35
Carrots	0.310	0.21	0.20
Orange peel	0.155	0.105	0.10
**Total weight**	**33.899**	**22.985**	**21.45**

BF1: baby food 1; BF2: baby food 2 and BF3: baby food 3.

### 2.3 Approximate analysis

The protein content in BF1, BF2, and BF3 was determined using the Kjeldahl method as described by Nelson and Sommers (1973). Crude fat and fiber contents were estimated using Soxhlet method Prosky method (Prosky et al., 1985), respectively. Moreover, mineral analysis was conducted with an atomic absorption spectrophotometer (Hitachi model 170–10) following the AOAC guidelines (2003).

### 2.4 Vitamin content analysis

Water-soluble vitamins in BF1, BF2, and BF3 were analyzed using HPLC (Agilent 1260 series, Agilent Technologies, Santa Clara, California, USA) with a C18 column (4.6 mm × 250 mm i.d., 5 μm). The mobile phase consisted of 0.01 % trifluoroacetic acid in water (pH 2.9) (A) and methanol (B), with a linear gradient as follows: 0 min (95 % A) at 1 ml/min, 0–4 min (95 % A) at 0.5 ml/min, 4–10 min (2 % A) at 0.6 ml/min, and 10–15 min (95 % A) at 1 ml/min. The multi-wavelength detector was set at 280 nm, and the injection volume was 10 μL per sample.

For fat-soluble vitamins, a separate HPLC (Agilent 1260 series) analysis was conducted using an Eclipse Plus C8 column (4.6 mm × 250 mm i.d., 5 μm). The mobile phase was a mixture of methanol and acetonitrile (65:35 *v*/v) at a flow rate of 1 ml/min. The diode array detector was set at 295 nm, and the fluorescence detector was set at 290 nm excitation and 330 nm emission. The injection volume was 10 μL per sample, with the column temperature maintained at 40 °C (Cortés-Herrera et al., 2019).

### 2.5 Amino acids composition

The amino acid composition was determined by mixing 1 g of BF1, BF2, or BF3 with 5 ml of water and 5 ml of hydrochloric acid (final concentration 6 M). The mixture was heated at 100 °C for 24 h and then filtered. Subsequently, 1 ml of the filtrate was injected into a High-Performance Liquid Chromatography (HPLC) system. HPLC analysis was performed using an Agilent 1260 series with an Eclipse Plus C18 column (3.0 mm × 150 mm i.d., 3.5 μm) as described by Serb (2013). The mobile phase comprised buffer (sodium phosphate dibasic and sodium borate) at pH 8.2 (A) and acetonitrile: methanol: water (45:45:10) (B), with a flow rate of 0.64 ml/min (Søren & Johansen, 2013).

### 2.6 Fatty acids composition

By converting the oils into their methyl esters (FAMEs) for gas chromatography (GC) analysis, with adjustments in accordance with Firestone (2009), the fatty acid composition of the oils was measured. 0.5 ml of n-hexane was used to dissolve the oils, and 100 μL of methanolic KOH (2 M) was added to turn the oils into FAMEs. The liquid was given time to settle after adding 2 M of hydrochloric acid until the methyl orange indicator turned pink. The organic layer was then injected into an Agilent 7890 GC (Agilent Technologies, Santa Clara, CA) fitted with a flame ionization detector using 10 μL of it. A stainless-steel column (30 m × 0.25 mm) packed with 70 % cyanopropyl polysilphenylene siloxane was used. Following sample injection, the oven temperature was maintained at 100 °C and subsequently raised to 225 °C at a rate of 5 °C/min. The temperatures of the injector and detector were adjusted to 260 °C and 280 °C, respectively. With an injection volume of 1 μL and a split ratio of 1:100, helium was utilized as the carrier gas, flowing at a rate of 3 ml/min. Fatty acid standards, such as myristic, palmitic, stearic, oleic, linoleic, linolenic, and arachidic acids (Sigma Aldrich, UK), were used to calculate the concentration of FAMEs in the samples.

### 2.7 Total phenolic contents (TPC)

The total phenolic content of BF1, BF2 and BF3 were determined spectrophotometrically by a T80 uv/vis spectrophotometer (PG Instrument Ltd. UK) using the Folin–Ciocalteu method. The results were expressed as mg gallic acid equivalent/g dry sample (Singleton & Rossi, 1965).

### 2.8 Total flavonoid contents (TFC)

Total flavonoid content of BF1, BF2 and BF3 was measured using the aluminium chloride colorimetric method (Zhishen et al., 1999). TFC was expressed as mg quercetin equivalent/g dry sample.

### 2.9 Antioxidant activity

The DPPH assays were carried out in accordance with Awad et al. (2020). The outcomes were contrasted with l-ascorbic acid's action. In summary, 3.5 ml of DPPH (0.06 mM in methanol) solution was mixed with 100 μL of each BF1, BF2, and BF3 (10 mg/ml in methanol) and left to incubate for 30 min in the dark. At 516 nm, the color changed from deep violet to pale yellow. Methanol was utilized as a control instead of DPPH and the sample. The following formula was used to determine the scavenging activity (%):

(1) $$\text{Scavenging}\%=1-\frac{\left(A\;\text{sample}-A\;\text{sample blank}\right)}{A\;\text{control}}*100$$

IC_50_ (half maximal inhibitory concentration) value is the concentration of the sample that can scavenge 50 % of DPPH free radical in DPPH free radical scavenging method.

### 2.10 Physical properties of the baby food formulations

#### 2.10.1 Color analyses

The color parameters of BF1, BF2, and BF3 were measured using a colorimeter (Smart Color Pro, Egypt Advanced Automation Systems).

#### 2.10.2 Texture profile analyses

BF1, BF2, and BF3 underwent texture profile analyses (TPA) utilizing a texture analyzer (Texture Pro CT V1.2, AMETEK Brookfield, 11 Commerce Blvd., Middleboro, MA, USA) in accordance with the procedure outlined by Bourne (2002). The TPA apparatus included a 57 mm diameter flat plate probe fixed to a moving crosshead, along with a load cell capable of supporting between 50 and 500 kg. Using a metal borer, cylindrical samples were made at 4–6 °C and then covered in plastic stretch film. After being removed from the surface at least 1 cm below, the samples were kept at 25 °C for around 30 min or until they stabilized at 19 ± 1 °C. A thermocouple was used to measure the specimens' core temperatures. The crosshead speed was set to 50 mm/min, the chart speed to 200 mm/min, and the compression ratio was set to 80 % from the sample's original height in two compressions. The TPA curve and data were used to calculate the texture profile characteristics, which include hardness, adhesive forces, adhesiveness, resilience, springiness, and springiness index.

### 2.11 Sensory evaluation

At Food Technology Department of the Arid Lands Cultivation Research Institute, SRTA-City, Alexandria, Egypt, ten women (average age = 35) were recruited as subjects in a panel test on fresh BF1, BF2, and BF3 formulations. The test was carried out as previously described by Abd El-Aziz et al., 2024, the following factors were assessed on a scale of 1 to 9: color, odor, taste, texture, appearance, and overall acceptability. One indicates extreme dislike, two indicate great dislike, three indicate moderate dislike, four indicate mild dislike, five indicate neither dislike nor like, six indicate slight like, seven indicate moderate like, eight indicate great like, and nine indicate extreme like. The panelists willingly accepted and consented to be involved in the sensory study and use their information. For this study, no ethical permission was required to carry out the sensory evaluation.

### 2.12 Statistical analysis

Version 16.0 of the IBM SPSS software program was used to analyze the obtained data (Kirkpatrick & Feeney, 2013). The terms mean and standard deviation was used to describe quantitative data. The F-test (ANOVA) was used to compare the various inhibitors under study for normally distributed data. The *p*-value (*p* < 0.05) was used to assess the importance of the obtained results (Kotz et al., 2006).

## 3 Results and discussion

### 3.1 Proximate composition

In this study, three different formulations of baby formula, BF1, BF2, and BF3, were analyzed for their proximate composition, mineral, and vitamin profiles on a dry weight basis (Table 2). Protein content varied notably among the formulations, with BF1 containing the highest amount at 4.63 ± 0.14 g/100 g, followed by BF2 (3.93 ± 0.21 g/100 g), and BF3 (1.05 ± 0.13 g/100 g). The protein content of the developed complementary foods contributes significantly toward meeting infants' daily requirements. A 50 g serving of BF1and BF2 could cover up to 25.84 % and 21.93 % respectively of the recommended daily intake of protein for infants aged 6–12 months. This discrepancy could impact the growth and development of infants, as protein is crucial for muscle and tissue formation (Xiong et al., 2023). Similarly, crude fat content showed substantial differences, with BF2 having the highest amount (1.98 ± 0.11 g/100 g), followed by BF1 (0.19 ± 0.02 g/100 g), and BF3 (0.13 ± 0.00 g/100 g). Results showed that lipid content in BF1, BF2 and BF3, can provide approximately 0.31 %, 3.19 % and 0.21 % respectively of the daily recommended intake for infants, for 50 g of each diet. Lipids play a crucial role in the diet of infants due to their multifaceted benefits. Firstly, they serve as a primary source of energy. Additionally, lipids providing insulation against heat loss and cushioning for vital organs. Moreover, they aid in the absorption of fat-soluble vitamins such as A, D, E, and K, essential for various physiological functions. Furthermore, lipids supply vital fatty acids necessary for normal brain development, healthy skin and hair, proper eye development, and bolstering the body's resistance against infections and diseases (Degrassi et al., 2023; Kleinman, 2004). Interestingly, BF3 exhibited significantly higher levels of crude fiber (23.50 ± 1.95 g/100 g) compared to BF1 (5.44 ± 0.23 g/100 g) and BF2 (3.01 ± 0.13 g/100 g). No dietary fiber is present in breast milk, and throughout the first six months of life, newborns typically do not eat any fiber. Fiber intake rises when complementary items are added to the diet (Agostoni et al., 1995). High fiber content in infant formula may not be ideal, as infants have limited digestive capabilities for fiber (Bakshi et al., 2023). The fiber levels in the formulations BF1and BF2 fall within the acceptable limits reported for complementary foods and support digestive health without contributing to constipation or reducing nutrient absorption. Also, total sugars content was also variable among the formulations, with BF1 containing the highest amount (9.35 ± 0.24 g/100 g), followed by BF3 (6.86 ± 0.35 g/100 g), and BF2 (5.28 ± 0.16 g/100 g). Excessive sugar intake in infants has been associated with various health issues, including obesity and dental problems (Awad et al., 2023). The observed variations in sugar content among the formulations contradict the recommendations outlined in the Dietary Guidelines for Americans and the American Heart Association, which advise that children under the age of two should avoid added sugars (Vos et al., 2017). There are various processing technologies such as enzymatic hydrolysis to modify carbohydrate could be applied to reduce the overall sugar content in the final product.

**Table 2:** Proximate composition of new baby foods (on dry weight basis).

Constituents (g/100 g)	BF1	BF2	BF3
Protein	4.63 ± 0.14^a^	3.93 ± 0.21^b^	1.05 ± 0.13^c^
Crude fat	0.19 ± 0.02^b^	1.98 ± 0.11^a^	0.13 ± 0^b^
Crude fiber	5.44 ± 0.23^b^	3.01 ± 0.13^c^	23.50 ± 1.95^a^
Total sugars	9.35 ± 0.24^a^	5.28 ± 0.16^c^	6.86 ± 0.35^b^
Ash	2.35 ± 0.25^b^	2.88 ± 0.12^b^	3.46 ± 0.13^a^
*Minerals (mg/100 g)*			
Fe	0.10 ± 0^b^	0.14 ± 0^a^	0.10 ± 0^b^
Na	58.11 ± 2.22^b^	33.66 ± 1.51^c^	101.76 ± 2.05^a^
Ca	67.68 ± 1.01^a^	24.58 ± 2.4^c^	36.73 ± 0.23^b^
Co	1.21 ± 0.11^a^	0.00086 ± 0^b^	0.00037 ± 0^b^

BF1: baby food 1; BF2: baby food 2 and BF3: baby food 3. Represented data are the means of *n* = 3 ± SD. The mean values indicated in the same rows within variable with different superscripts (^a, b^ and ^c^) were significantly different (*p* < 0.05).

Furthermore, mineral composition differed significantly among the formulations. For example, BF3 exhibited substantially higher ash content (3.46 ± 0.133 g/100 g) compared to BF1 (2.35 ± 0.25 g/100 g) and BF2 (2.88 ± 0.12 g/100 g). Ash content represents the inorganic residue left after complete combustion of organic matter and includes minerals such as calcium, phosphorus, and magnesium (Xu et al., 2024). The mineral content analysis of BF1, BF2, and BF3 revealed significant variations. Iron (Fe) content was observed to be highest in BF2 at 0.137 mg/100 g, followed by BF1 at 0.1003 mg/100 g, and BF3 at 0.1026 mg/100 g. Sodium (Na) content was notably higher in BF3 at 101.76 mg/100 g compared to BF1 (58.11 mg/100 g) and BF2 (33.6675 mg/100 g). Calcium (Ca) content showed variations with BF1 exhibiting the highest concentration at 67.68 mg/100 g, followed by BF3 at 36.73 mg/100 g, and BF2 at 24.585 mg/100 g. Cobalt (Co) content was minimal across all samples. These minerals are essential for bone development and overall growth in infants (Xu et al., 2024). By comparing the mineral composition of our baby food formulations with a commercially available complementary food, Nestlé Cerelac, distinct differences in mineral concentrations are observed. Specifically, the iron content in our formulations ranged from 0.1003 to 0.137 mg/100 g, whereas Nestlé Cerelac contains approximately 11 mg/100 g. Similarly, calcium levels in our products varied between 24.585 and 67.68 mg/100 g, while Nestlé Cerelac provides 602 ± 0.02 mg/100 g. For sodium, our formulations contained 33.6675 to 101.76 mg/100 g, compared to 143 ± 3 mg/100 g in Nestlé Cerelac (Oyegoke et al., 2020). These differences can be largely attributed to the intentional fortification practices employed in commercial infant food manufacturing. Products like Cerelac are fortified with essential micronutrients such as iron and calcium to meet the dietary recommendations for infants and toddlers, ensuring nutritional adequacy regardless of natural ingredient variability.

### 3.2 Vitamins Content

The analysis of BF1, BF2, and BF3, revealed significant variations in vitamin content (Table 3). Vitamin A content ranged from 71.5 μg/100 g in BF1 to 115 μg/100 g in BF2, while BF3 exhibited a lower concentration at 47.2 μg/100 g. Vitamin D3 content varied from 7.4 μg/100 g in BF1 to 4.45 μg/100 g in BF3. Also, BF1 had 4.5 μg/100 g of vitamin E, whereas BF2 had 12 μg/100 g and BF3 had 8.6 μg/100 g. Vitamin C content ranged from 2.69 mg/100 g in BF1 to 1.45 mg/100 g in BF2, with BF3 at 2.7 mg/100 g. Notably, BF1 exhibited higher levels of vitamin B1 at 34.1 mg/100 g compared to 12.6 mg/100 g in BF2 and 58.1 mg/100 g in BF3. Vitamin B9 content varied significantly, with BF1 containing 14,500 μg/100 g, BF2 containing 1260 μg/100 g, and BF3 containing 3620 μg/100 g. BF1 also had higher levels of vitamin B12 at 1830 μg/100 g compared to 1810 μg/100 g in BF2. When comparing the vitamin composition of the newly formulated baby food samples (BF1, BF2, and BF3) with a commercial standard (Nestlé Cerelac), notable differences emerge in both deficiencies and advantages. In terms of vitamin A, which is essential for visual development and immune competence, all three formulations demonstrated significantly lower content than Cerelac (1300.45 μg/100 g), with BF2 containing the highest among them (115.0 μg/100 g), covering only approximately 9 % of the commercial benchmark. This underlines the need for fortification or incorporation of vitamin A-rich ingredients to meet the nutritional requirements of infants. Conversely, BF1 and BF2 provided remarkably elevated levels of vitamin B12 (1830 and 1810 μg/100 g, respectively), dramatically exceeding Cerelac's content (3.0 μg/100 g). This exceptional difference, over 600-fold, positions these formulations as highly advantageous for supporting neurological development, particularly beneficial for populations with limited access to animal-source foods. Similarly, BF1 exhibited an extraordinary concentration of folate (vitamin B9), reaching 14,500 μg/100 g, compared to just 30 μg/100 g in Cerelac. Folate is critical for DNA synthesis, red blood cell formation, and brain development, reinforcing BF1's potential as a functional food for early-life nutrition. Vitamin E content was also favorable in BF2 (12.0 μg/100 g), outperforming Cerelac (5.0 μg/100 g), while BF1 and BF3 exhibited comparable antioxidant potential (4.5 and 8.6 μg/100 g, respectively). However, the formulations were substantially lower in vitamin C (ranging from 1.45 to 2.70 mg/100 g) compared to Cerelac's 64.69 mg/100 g. Given vitamin C's role in immune function and enhancing iron absorption, this result suggests a practical opportunity for improvement through the inclusion of citrus fruits or other ascorbic acid-rich ingredients. (Dieterich et al., 2013; Oyegoke et al., 2020).

**Table 3:** Vitamins content of new baby food formulas.

Vitamins/100 g	BF1	BF2	BF3
Vit. A (μg)	71.50 ± 1.25^b^	115.00 ± 2.13^a^	47.20 ± 1.61^c^
Vit. D3 (μg)	7.40 ± 0.14^a^	6.90 ± 0.09^b^	4.45 ± 0.13^c^
Vit. E (μg)	4.50 ± 0.05^c^	12.0 ± 0.32^a^	8.60 ± 0.24^b^
Vit. C (mg)	2.69 ± 0.04^a^	1.45 ± 0.02^b^	2.70 ± 0.11^a^
Vit. B1 (mg)	34.10 ± 1.01^b^	12.60 ± 0.09^c^	58.10 ± 0.87^a^
Vit. B2 (mg)	0.158 ± 0.02^b^	0.43 ± 0.01^a^	0
Vit. B9 (μg)	14,500 ± 1.74^a^	1260 ± 1.08^c^	3620 ± 1.25^b^
Vit. B12 (μg)	1830 ± 0.48^a^	1810 ± 0.36^b^	0

BF1: baby food 1; BF2: baby food 2 and BF3: baby food 3. Represented data are the means of n = 3 ± SD. The mean values indicated in the same rows within variable with different superscripts (a, b and c) were significantly different (*p* < 0.05).

### 3.3 Amino acids composition

Based on the analysis of amino acid content (Table 4), BF2 exhibited notably higher concentrations of essential amino acids compared to BF1 and BF3, particularly in methionine, phenylalanine, and leucine, with values of 241.06 μg/g, 476.41 μg/g, and 443.36 μg/g, respectively. Conversely, BF1 showed lower levels of essential amino acids, including tryptophan, in comparison to BF2 and BF3. Interestingly, BF3 displayed intermediate concentrations of amino acids between BF1 and BF2, indicating a relatively balanced profile. Notably, tryptophan content varied considerably among the formulas, with BF2 demonstrating the highest concentration at 980.24 μg/g, followed by BF3 at 508.07 μg/g, and BF1 at 0.82 μg/g. When compared to the reference amino acid patterns recommended for infants by the FAO/WHO (2022) and Arsenault and Brown (2017), BF2 meets or exceeds the required levels for several essential amino acids, particularly leucine, phenylalanine, and tryptophan, which are critical for protein synthesis and neurological development in infants. However, BF1's low tryptophan content suggests it may not fully meet the nutritional needs without supplementation. In this context, complementary feeding with other protein-rich foods such as dairy products, eggs, or legumes could help bridge the gap, ensuring a complete amino acid profile essential for optimal growth (Friedman, 2018). Overall, while BF2 appears most promising in terms of essential amino acid adequacy, integrating the complementary foods with a varied diet rich in diverse protein sources would ensure a balanced amino acid intake, particularly for formulas like BF1 that fall short in specific essential amino acids.

**Table 4:** Amino acid composition of new baby food formulations.

Amino acids (μg/g)	BF1	BF2	BF3
*Non-essential amino acids*			
Asparagine	384.81 ± 3.18^b^	211.26 ± 2.06^c^	454.54 ± 4.91^a^
Glutamic acid	345.08 ± 2.51^c^	487.24 ± 5.44^b^	607.51 ± 5.28^a^
Serine	112.95 ± 3.18^b^	155.77 ± 4.77^a^	82.22 ± 2.96^c^
Glycine	128.53 ± 0.92^b^	138.28 ± 2.01^a^	87.55 ± 0.71^c^
Proline	52.84 ± 1.36^c^	88.82 ± 3.02^a^	62.57 ± 1.97^b^
Arginine	237.28 ± 5.01^a^	153.44 ± 2.45^c^	190.82 ± 2.28^b^
Alanine	114.76 ± 2.88^b^	199.98 ± 1.21^a^	88.38 ± 2.06^c^
Tyrosine	45.43 ± 2.39^c^	82.6 ± 1.95^a^	70.28 ± 1.74^b^
Cystine	0	0	0
*Essential amino acids*			
Threonine	75.46 ± 2.13^b^	84.15 ± 1.36^a^	0
Histidine	16.50 ± 0.71^a^	15.63 ± 1.08^a^	11.73 ± 0.82^b^
Valine	99.36 ± 1.78^b^	99.74 ± 0.98^b^	111.71 ± 1.03^a^
Methionine	210.47 ± 1.57^b^	241.06 ± 1.42^a^	187.74 ± 0.86^c^
Phenylalanine	453.18 ± 3.54^b^	476.41 ± 4.27^a^	438.45 ± 2.34^c^
Isoleucine	101.47 ± 1.02^b^	108.59 ± 1.34^a^	87.45 ± 1.55^c^
Leucine	287.40 ± 2.88^b^	443.36 ± 3.21^a^	227.79 ± 0.97^c^
Lysine	82.78 ± 1.51^b^	79.27 ± 1.88^b^	105.21 ± 2.41^a^
Tryptophan	0.82 ± 0.06^c^	980.24 ± 2.85^a^	508.06 ± 4.05^b^

BF1: baby food 1; BF2: baby food 2 and BF3: baby food 3. Represented data are the means of n = 3 ± SD. The mean values indicated in the same rows within variable with different superscripts (a, b and c) were significantly different (*p* < 0.05).

### 3.4 Fatty acid composition

The results illustrated in Table 5 outlines the concentrations of fatty acids in three baby food samples (BF 1, BF 2, and BF 3), measured in milligrams per kilogram (mg/kg) of lipid content. Among omega-6 fatty acids, linoleic acid (LA) was significantly abundant, with BF3 showing the highest concentration at 3016.59 mg/kg, followed by BF2 at 244.83 mg/kg. However, gamma-linolenic acid (GLA) was absent in BF2 and BF3 but BF1 contain 12 mg/kg. In terms of omega-3 fatty acids, alpha-linolenic acid (ALA) was present in all samples, with BF2 demonstrating the highest concentration at 115.77 mg/kg. Conversely, docosahexaenoic acid (DHA) was undetectable in all samples, indicating a potential deficiency in this crucial fatty acid. Eicosapentaenoic acid (EPA) was present in BF3 at the highest concentration of 20.89 mg/kg while it was 12.0 mg/kg in BF1. Among non-essential fatty acids, caproic acid, caprylic acid, and Palmitic acid were notably present across all samples. Notably, pentadecylic acid was significantly higher in BF1 compared to BF2 and BF3, highlighting differences in non-essential fatty acid composition among the samples. The amount of linoleic acid in breast milk is about 5.6 g/l, whereas infant formulae currently include 3.3–28.6 g/l (Institute of Medicine, 2005). Furthermore, infant formulae supply 0 to 0.67 g/l of Omega-3 polyunsaturated fatty acids (including docosahexaenoic acid and α-linolenic acid) whereas breast milk supplies about 0.63 g/l (Institute of Medicine, 2005). More critically, DHA was entirely absent in all three formulations, despite its well-established role in supporting cognitive development, visual acuity, and neural maturation in infants (Hu et al., 2024). The combined absence of GLA, EPA, and DHA in BF2, and partial absence in other formulations, reveals a nutritional gap that may limit the optimal neurodevelopmental and immunological benefits of these foods. To address this limitation, we recommend the incorporation of long-chain polyunsaturated fatty acids (LCPUFAs)—particularly DHA and EPA—via fortification strategies commonly used in the infant nutrition industry. Algal oil and microencapsulated fish oil are both safe, bioavailable, and sensory-neutral options widely adopted in commercial infant formula production (Zhang et al., 2024). Algal oil offers a sustainable, vegetarian-friendly source of DHA, while microencapsulation enhances oxidative stability and reduces off-flavors. Inclusion of these LCPUFAs, alongside functional levels of GLA from sources such as evening primrose or borage oil, could significantly enhance the developmental value of the formulated baby foods, bringing them in line with international guidelines for essential fatty acid intake during early childhood. Consequently, manufacturers of infant formulas add blends of vegetable oils, which are high in linoleic acid, to improve essential fatty acid content (Kleinman, 2004).

**Table 5:** Fatty acid composition of new baby food formulations (mg/kg).

Fatty acid (mg/Kg)	Lipid numbers	BF1	BF2	BF3
Essential fatty acids				
*Omega- 6*				
Linoleic acid (LA)	C18:2 (c9, c12)	12.00 ± 0.36^c^	244.83 ± 1.04^b^	3016.59 ± 2.71^a^
Gamma-Linolenic acid (GLA)	C18:3 (c6, c9, c12)	12.00 ± 0.08^a^	0	
*Omega- 3*				
Alpha-Linolenic acid (ALA)	C18:3 (c9, c12, c15)	12.00 ± 0.05^b^	5.82 ± 0.11^c^	115.77 ± 2.02^a^
Docosahexaenoic acid (DHA (	C22:6 (c4, c7, c10, c13, c16, c19)	0	0	0
Eicosapentaenoic acid (EPA)	C20:5 (c5, c8, c11, c14, c17)	12.00 ± 0.14^b^	0	20.89 ± 0.23^a^
Non - essential fatty acids				
*Omega- 9*				
Oleic acid	C18:1 (c9)	24.00 ± 0.08^a^	0	0
*Other non - essential fatty acids*				
Caproic acid	C6:0	24.00 ± 1.03^c^	228.75 ± 1.51^b^	262.37 ± 1.78^a^
Caprylic acid	C8:0	24.00 ± 0.88^c^	128.40 ± 1.87^b^	205.75 ± 2.24^a^
Pentadecylic acid	C15:0	12.00 ± 1.01^c^	4446.98 ± 5.66^a^	3697.73 ± 5.04^b^
Palmitic acid	C16:0	36.00 ± 0.92^c^	4361.82 ± 6.32^b^	5492.28 ± 6.94^a^

BF1: baby food 1; BF2: baby food 2 and BF3: baby food 3. Represented data are the means of n = 3 ± SD. The mean values indicated in the same rows within variable with different superscripts (a, b and c) were significantly different (*p* < 0.05).

### 3.5 Phytochemical analysis and antioxidant activity of baby food formulations

The phytochemical analysis and assessment of antioxidant activity for the three baby food formulations (BF1, BF2, and BF3) are presented in Table 6. Among the samples, BF3 exhibited the highest total phenolic content (TPC) at 83.87 ± 1.80 mg/g, followed by BF2 (75.10 ± 1.76 mg/g) and BF1 (74.77 ± 0.85 mg/g), suggesting considerable antioxidant potential due to phenolic compound richness (Orsavová et al., 2023). This aligns with the study by Bana and Gupta (2015), where a Gorgon nut–based infant formula showed a TPC of 16.71 μg GAE/mg extract and demonstrated appreciable antioxidant activity. Regarding total flavonoid content (TFC), BF1 had the highest value (31.36 ± 1.18 mg/g), followed closely by BF3 (30.91 ± 1.43 mg/g) and BF2 (29.75 ± 1.47 mg/g). These values are notably higher than the 85.26 μg RE/g reported in the formulation by Bana and Gupta, possibly due to differences in ingredient composition and processing methods such as lyophilisation. This suggests that our naturally developed formulations, especially BF1 and BF3, are strong contributors to dietary flavonoids and thus offer functional health benefits. In terms of antioxidant activity assessed via DPPH assay, BF1 showed the lowest IC50 value (480.63 ± 4.97 μg/ml), indicating the highest radical scavenging activity, followed by BF3 (838.99 ± 2.08 μg/ml) and BF2 (1356.00 ± 5.57 μg/ml). This result contrasts with Bana and Gupta's F1 formulation, which exhibited 81.35 % DPPH inhibition, demonstrating strong antioxidant potential. These variations may stem from ingredient origin and synergistic effects among phytochemicals in different matrices. Such findings highlight the promise of integrating antioxidant-rich complementary foods in infant diets to provide not only basic nutrition but also protective health effects against oxidative stress-related conditions (Ajayi et al., 2023; Orsavová et al., 2023). Furthermore, the inclusion of phenolic and flavonoid-rich ingredients in infant formulations, as evidenced in both our study and previous reports (Almeida et al., 2021; Bana & Gupta, 2015), supports the development of nutritionally enhanced weaning foods with functional benefits.

**Table 6:** Phytochemical analysis and antioxidant activity of new baby food formulas.

Phytochemicals	BF1	BF2	BF3
Total phenolic content (TPC) (mg/g)	74.77 ± 0.85^b^	75.10 ± 1.76^b^	83.87 ± 1.80^a^
Total flavonoid content (TFC) (mg/g)	31.36 ± 1.18^a^	29.75 ± 1.47^a^	30.91 ± 1.43^a^
DPPH (IC_50_ μg/ml)	480.63 ± 4.97^c^	1356.00 ± 5.57^a^	838.99 ± 2.08^b^

BF1: baby food 1; BF2: baby food 2 and BF3: baby food 3. Represented data are the means of *n* = 3 ± SD. The mean values indicated in the same rows within variable with different superscripts (^a, b^ and ^c^) were significantly different (*p* < 0.05).

### 3.6 Physical properties of the baby food

#### 3.6.1 Color analyses

The color parameters of the new baby food were examined to understand its visual characteristics (Table 7). The L* value, representing lightness, showed a descending trend from BF1 (76.39) to BF3 (45.79), indicating darker crusts in the latter samples. The a* value, indicating redness (+a*) or greenness (−a*), varied notably across the samples, with BF2 displaying a negative value (−15.23), indicating a greenish hue, while BF1 and BF3 showed positive values (0.50 and 12.92, respectively), suggesting reddish tones. The b* value, representing yellowness (+b*) or blueness (−b*), was negative for all samples, with BF3 exhibiting the lowest value (−93.42), indicating a bluish hue, followed by BF1 (−74.25) and BF2 (−51.86). So that, BF3 was the most appealing in terms of color, based on the sensory evaluation and colorimetric measurements (highest a* and darkest L* values).

**Table 7:** Color parameters of the crust of new baby food formulations.

Color parameter	BF1	BF2	BF3
L*	76.39 ± 2.52^a^	60.97 ± 1.84^b^	45.79 ± 1.77^c^
a*	0.50 ± 0.03^b^	−15.23 ± 1.08^c^	12.92 ± 1.01^a^
b*	−74.25 ± 2.52^b^	−51.86 ± 2.65^a^	−93.42 ± 3.78^c^

BF1: baby food 1; BF2: baby food 2 and BF3: baby food 3. Represented data are the means of n = 3 ± SD. The mean values indicated in the same rows within variable with different superscripts (a, b and c) were significantly different (*p* < 0.05).

L: is lightness; a: is redness; b: is yellowness.

These variations in color parameters among the crusts of the baby food samples may result from differences in ingredients, processing methods, or storage conditions, highlighting the importance of considering visual appeal alongside nutritional content in infant food formulations (Pathare et al., 2013). Investigation of color stability during storage and shelf-life could provide insights into product quality maintenance over time, ensuring consistent consumer satisfaction.

#### 3.6.2 Texture profile analyses

The finding presented in Table 8 display the texture properties of the new baby foods, including hardness, deformation at hardness, hardness work, total work, adhesion force, and adhesiveness, for three different samples (BF1, BF2, and BF3). Hardness, measured in grams (g), indicates the force required to compress the baby food samples. BF3 exhibited the highest hardness at 310 g, followed by BF2 at 255 g and BF1 at 157 g. These results suggest that BF3 had the firmest texture, while BF1 was the softest. Deformation at hardness, measured in millimeters (mm), refers to the amount of deformation the sample undergoes at the specified hardness. BF1 and BF2 showed similar deformation values at 4 mm and 3.99 mm, respectively, while BF3 exhibited the same deformation as BF2. Hardness work, total work, adhesion force, and adhesiveness provide insights into the energy required for compression, overall work done during compression, stickiness, and the extent of stickiness, respectively (Bourne, 2002). BF2 required the highest hardness work at 6.2 mJ, followed by BF3 at 5.5 mJ and BF-1 at 3.8 mJ. Similarly, BF2 showed the highest total work at 6.3 mJ, followed by BF3 at 5.7 mJ and BF1 at 3.8 mJ. These results indicate that BF2 required the most energy for compression and exhibited the highest overall work done during compression. Regarding adhesion properties, BF1 and BF2 demonstrated similar adhesion forces of 56 g and 55 g, respectively, while BF3 showed a lower adhesion force of 26 g. Adhesiveness followed a similar trend, with BF1 having the highest adhesiveness at 2.1 mJ, followed by BF2 at 1.7 mJ and BF3 at 0.7 mJ. Based on the results, BF2 appears to provide the most favorable overall texture profile, particularly when aiming for a balanced combination of firmness and ease of handling, without excessive stickiness. It demonstrates high structural integrity—evidenced by the highest values for both hardness work and total work—moderate adhesion characteristics, and consistent deformation comparable to the other samples, indicating a uniform and stable texture. In general, texture is a critical aspect of baby foods, impacting consumer acceptance and product quality. The optimization of texture properties in baby formula is critical for meeting the sensory preferences and nutritional needs of infants. Cappellotto and Olsen (2021) emphasizes that texture influences the development of oral motor skills, which are essential for chewing, swallowing, and speech. Texture plays a pivotal role in shaping infants' acceptance of foods and the development of essential oral motor skills. As reviewed by Chow et al. (2024), infants demonstrate a natural preference for smooth and soft textures during early complementary feeding stages, while gradual exposure to more complex textures—such as lumpy or firm consistencies—promotes maturation of chewing abilities and safe swallowing reflexes.

**Table 8:** Texture properties of new baby food formulas.

Texture parameter	BF1	BF2	BF3
Hardness cycle1 (g)	157.00 ± 0.71^c^	255.00 ± 0.33^b^	310.00 ± 1.04^a^
Deformation at hardness (mm)	4.00 ± 0.01^a^	3.99 ± 0.05^a^	3.99 ± 0.04^a^
Hardness work cycle 1 (mJ)	3.80 ± 0.07^c^	6.20 ± 1.01^a^	5.50 ± 0.07^b^
Total work cycle 1 (mJ)	3.80 ± 0.15^c^	6.30 ± 1.12^a^	5.70 ± 0.94^b^
Adhesion force (g)	56.00 ± 1.14^a^	55.00 ± 1.89^a^	26.00 ± 0.46^b^
Adhesiveness (mJ)	2.10 ± 0.22^a^	1.70 ± 0.08^b^	0.70 ± 0.01^c^

BF1: baby food 1; BF2: baby food 2 and BF3: baby food 3. Represented data are the means of n = 3 ± SD. The mean values indicated in the same rows within the variable with different superscripts (a, b and c) were significantly different (*p* < 0.05).

### 3.7 Sensory Evaluation

**Fig. 3:**
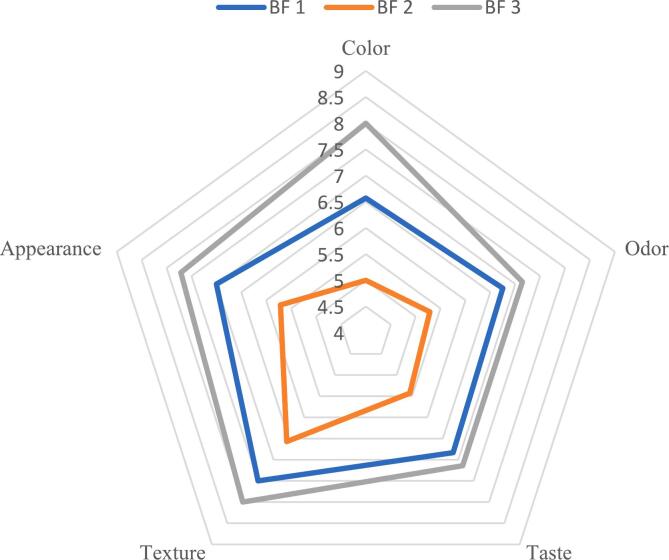
Sensory evaluation of new baby food formulations. BF1: baby food 1; BF2: baby food 2 and BF3: baby food 3. (1) Dislike extremely; (2) dislike very much; (3) dislike moderately; (4) dislike slightly; (5) neither like nor dislike; (6) like slightly; (7) like moderately; (8) like very much and like extremely (9).

The sensory evaluation of the new baby formula products, as depicted in Fig. 3, revealed color as the most influential parameter, with panelists preferring BF3, followed by BF1 and BF2, scoring 8, 6.57, and 5, respectively. These preferences align with our color analyses (Table 7), particularly regarding the redness color of BF3. The sensory assessment indicated BF3 as the most acceptable, garnering the highest score among the new baby formula variants. Notably, BF3, formulated with potatoes and mushrooms, emerged as the preferred option, potentially due to the texture, taste, and appearance enhancements facilitated by starch and fiber. Furthermore, BF3's popularity could be attributed to its natural colors derived from beets, carrots, and orange peel. Recognizing the significance of sensory perception in maintaining consumer satisfaction and bolstering the wholesome image of baby foods, sensory measurements serve as vital steps in quality and consistency evaluations (Drake, 2007).

## 4 Conclusions

The current investigation into three distinct baby food formulations, BF1, BF2, and BF3, revealed significant disparities in their nutritional compositions and sensory acceptance. BF1 expresses higher protein and sugar contents than BF2 and BF3. Additionally, BF2 exhibited high fat content compared to BF1 and BF3. Furthermore, BF3 showed the highest content in crude fiber and ash. Notably, BF2 emerged as particularly rich in vitamin D3, vitamin E, and essential amino acids, which are vital for infant growth. Conversely, BF1 displayed elevated levels of vitamin A, vitamin B12, linoleic acid, and antioxidant activity. Interestingly, despite these differences, sensory evaluation favoured BF3 followed by BF1 and BF2. These findings underscore the importance of carefully considering both nutritional content and sensory attributes when designing baby food formulations. Further research is warranted to elucidate the implications of these variations on infant health and development.

## CRediT authorship contribution statement

**Nourhan M. Abd El-Aziz:** Writing – original draft, Software, Resources, Methodology, Formal analysis, Data curation, Conceptualization. **Hanem M.M. Mansour:** Writing – original draft, Visualization, Methodology, Formal analysis, Data curation, Conceptualization. **Marwa R. Elbakatoshy:** Writing – original draft, Visualization, Validation, Investigation, Formal analysis, Data curation, Conceptualization. **Taha Mehany:** Writing – original draft, Visualization, Validation, Software, Resources, Methodology, Investigation, Data curation, Conceptualization. **Oscar Zannou:** Writing – review & editing, Visualization, Validation, Project administration, Funding acquisition. **Reza Tahergorabi:** Writing – review & editing, Visualization, Validation, Project administration, Funding acquisition. **Mohamed G. Shehata:** Writing – review & editing, Visualization, Validation, Supervision, Software, Resources, Conceptualization.

## Declaration of competing interest

The authors declare that they have no known competing financial interests or personal relationships that could have appeared to influence the work reported in this paper.

## Data Availability

Data will be made available on request.
